# Supercritical carbon dioxide: putting the fizz into biomaterials

**DOI:** 10.1098/rsta.2005.1687

**Published:** 2005-11-30

**Authors:** John J.A. Barry, Marta M.C.G. Silva, Vladimir K. Popov, Kevin M. Shakesheff, Steven M. Howdle

**Affiliations:** 1 Tissue Engineering Group, School of Pharmacy, The University of NottinghamUniversity Park, Nottingham NG7 2RD, UK; 2 School of Chemistry, The University of NottinghamUniversity Park, Nottingham NG7 2RD, UK; 3 Institute of Laser and Information Technologies, Russian Academy of SciencesPionerskaya 2, Troitsk, Moscow Region 142092, Russia

**Keywords:** supercritical carbon dioxide, polymer scaffold foam

## Abstract

This paper describes recent progress made in the use of high pressure or supercritical fluids to process polymers into three-dimensional tissue engineering scaffolds. Three current examples are highlighted: foaming of acrylates for use in cartilage tissue engineering; plasticization and encapsulation of bioactive species into biodegradable polyesters for bone tissue engineering; and a novel laser sintering process used to fabricate three-dimensional biodegradable polyester structures from particles prepared via a supercritical route.

## 1. Scaffolds and supercritical carbon dioxide

The key to tissue engineering is to replace diseased or damaged tissues with an immunologically tolerant living/viable tissue that can grow with the patient (Sachlos & Czernuszka 2003). This involves either growing *in vitro* a tissue for transplantation or implanting all the necessary ingredients to create the tissue *in vivo*. Although both approaches may appear very different, they have the same requirements. These are:

- cells that will proliferate to create the tissue; these can be transplanted or from the surrounding tissue;
- growth factors that signal the cells to proliferate; and
- a method of delivery and stabilizing the cells in the defects; the vehicle for delivering cells is called a scaffold.

Scaffolds are crucial to tissue engineering strategies for a number of reasons; as a three-dimensional structure they provide volume fill, mechanical integrity and a surface that can provide chemical and architectural guidance for regenerating tissues (Goldstein & Moalli 2001). Research in this area has been dominated by developing new scaffold materials and new scaffold fabrication techniques (see Leong *et al*. 2003; Ma 2004 for review). Our research has focused on using carbon dioxide gas as a porogen to create three-dimensional polymeric structures that can be used as scaffolds.

**Figure 1:**
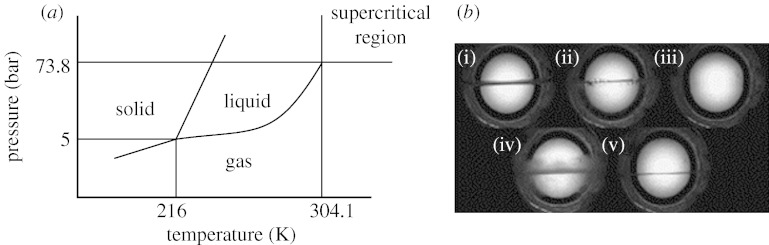
(*a*) Phase diagram for carbon dioxide, showing critical point and supercritical region (McHugh & Krukonis 1994). (*b*) A practical demonstration showing the creation of the single supercritical fluid phase (image (iii)) by taking liquid CO_2_ above its critical temperature and pressure (images (i) and (ii)); with subsequent lowering of temperature (and hence pressure) the process is reversed (images (iv) and (v)) (Quirk *et al*. 2004; Howdle 2004).

Carbon dioxide gas above a critical temperature (*T*_c_=304.1 K) and pressure (*P*_c_=73.8 bar) is said to be supercritical (figure 1). In this state the carbon dioxide is neither a gas nor a liquid but has properties of both. This is clearly demonstrated by the phase diagram in figure 1; changing the temperature and pressure changes the phase from solid to liquid to gas. However, at the critical point (the intersection of *T*_c_ and *P*_c_), the distinction between the liquid and gas phases disappears. The single fluid phase CO_2_ at this point (figure 1*b*(iii)) is said to be supercritical. Supercritical carbon dioxide (scCO_2_) is an attractive solvent for polymer processing, as it is inexpensive, non-toxic, non-flammable and its properties can be tuned through its density (Woods *et al*. 2004). More importantly, however, is the observation that some polymers when processed in scCO_2_ swell to create porous materials. This swelling or foaming is due to scCO_2_ being soluble in the polymers (see Cooper 2000 for review). The diffusion of scCO_2_ into a polymer matrix separates the polymer chains and lowers their resistance to chain rotation; this is called plasticization. The plasticization of polymers by scCO_2_ results in a depression of the glass transition temperature (*T*_g_). For example, the *T*_g_ of PMMA was found to be reduced to near room temperature at 12–15 wt% of CO_2_ absorbed (Goel & Beckman 1994*a*).

While in this highly plasticized state, the solubility of the gas in the polymer can be decreased by reducing the CO_2_ gas pressure to atmospheric pressure (Goel & Beckman 1994*a*). This drop in pressure is responsible for the occurrence of two physical events. First the sudden reduction in pressure leads to a decrease in the solubility of the gas in the polymer and this generates nuclei (or bubbles). These nuclei grow to form the pores in the foam. The porous structures become fixed by the second event. As CO_2_ leaves the polymer the *T*_g_ begins to rise again, eventually reaching a temperature close to the foaming equipment. The polymer then becomes glassy (vitrification) and the pores can grow no further and are said to be ‘locked in’ (Goel & Beckman 1994*a*; Mooney *et al*. 1996; Arora *et al*. 1998; Cooper 2000).

Nucleation (as described earlier) is usually accompanied with and competes with the diffusion of gas into pores. This diffusion of gas into pores results in pore growth. If the venting rate is high, then nucleation is rapid and there will be a large number of nucleation sites. Each pore will develop so fast that the diffusion effects will be negligible and the resultant structure will have a homogeneous or uniform pore size distribution. On the other hand, if nucleation is very slow, the pores nucleated first will be significantly larger than others due to greater diffusion of gas from the surrounding matrix, and the resultant structure would have wide dispersion in pore size (Alavi *et al*. 1999). Being able to control these two events is important in creating scaffolds suitable for tissue engineering and has been demonstrated by using different gases (CO_2_, N_2_ and He), changing the molecular weight of the polymer and manipulating processing conditions such as temperature, pressure and venting rate (Goel & Beckman 1994*a*,*b*; Park *et al*. 1995; Arora *et al*. 1998; Sheridan *et al*. 2000; Howdle *et al*. 2001).

**Figure 2:**
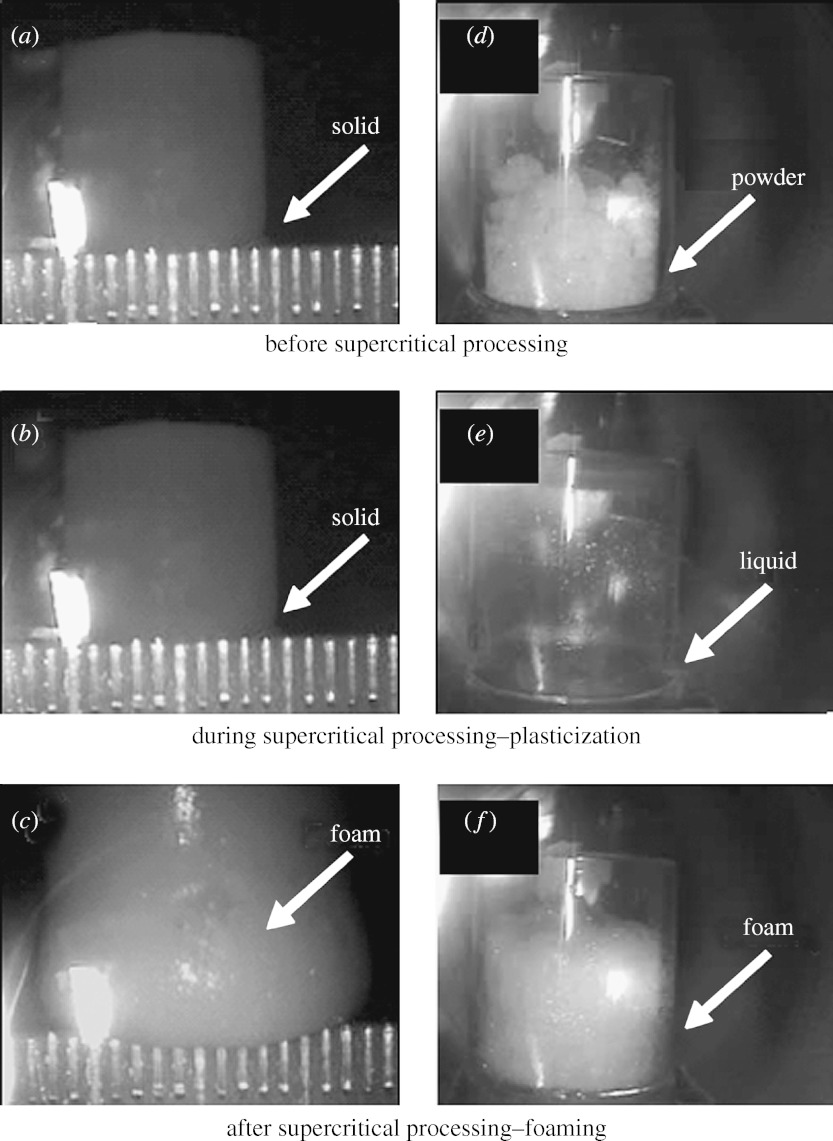
The *in situ* plasticization and foaming of amorphous polymers. Images are taken through a high-pressure vessel with a sapphire window. This plasticization and foaming can occur via two routes. The first route is shown in images (*a*)–(*c*), the polymer, although plasticized, remains structurally unchanged until nearly all the carbon dioxide gas has been vented and then the polymer foams. In these images, the internal diameter of the vessel has been restricted by the incorporation of a graduated scale (mm markings) (Barry *et al*. in press *a*). The second route is shown in images (*d*)–(*f*), the polymer powder is plasticized forming a polymer gas solution which foams when all the carbon dioxide gas has been vented (Quirk *et al*. 2004).

The depression of the polymer *T*_g_ has been captured *in situ* using a view cell (figure 2). The outcome of plasticization can be different in that some polymers such as the methacrylate-based glassy polymers (§2*a*) retain their structural integrity under supercritical conditions, while others such as poly(d,l-lactide) form a polymer/gas solution (§2*b*). We have prepared scaffolds from both polymer types.

## 2. Tetrahydrofurfuryl methacrylate scaffolds

The heterocyclic, tetrahydrofurfuryl methacrylate (THFMA) has been shown to have some intriguing properties. It is a methacrylate with a large pendent group that has good miscibility with other polymeric systems, which is thought to be due to the presence of additional moieties such as the ether oxygen atoms in the pendent groups (Lee *et al*. 1992).

When blended with poly(ethyl methacrylate) (PEMA), a glassy semi-interpenetrating network is formed that exhibits both low shrinkage and a low exotherm on polymerization, as well as a low glass transition temperature compared to other methacrylates (Patel *et al*. 1987). More interestingly, however, PEMA/THFMA blends have been shown to remain rigid over a long time period, while having a high water uptake (more than 70 wt% in 3 years; Hutcheon *et al*. 1998). The water uptake behaviour is composed of two stages, an initial rapid Fickian controlled stage that is followed by a long-term slow rate of uptake, which is dominated by a clustering effect (Riggs *et al*. 1999*a*). This anomalous uptake behaviour, which is dependent on the osmolarity of the external solution (Sawtell *et al*. 1997), has been attributed to the THFMA component in the composition (Patel *et al*. 2001*a*).

Blend systems formed by mixing THFMA with an isoprene–styrene copolymer elastomer (SIS) are currently being evaluated for soft prosthetic applications (Nazhat *et al*. 2001). The mechanical properties of these materials can be tailored by altering the composition of the SIS and THFMA in the blend. These compositions also demonstrate a two-stage water uptake, attributable to the THFMA component of the blend (Nazhat *et al*. 2001).

### (a) PEMA/THFMA

Bhusate & Braden were first to describe a methacrylate polymer system that involved the mixing of a powdered polymer constituent (PEMA) that initiates the polymerization of a monomeric ester (THFMA) in the liquid form (Bhusate & Braden 1985). Residual benzoyl peroxide initiator, still present in the PEMA, reacts with a tertiary amine (*N*,*N-*dimethyl-*p*-toluidine), resulting in the formation of free radicals, capable of initiating polymerization of the THFMA monomer (Bhusate & Braden 1985; Riggs *et al*. 2000). Thus far PEMA/THFMA has been investigated for potential uses in dental applications, ion, protein and drug release (Pearson *et al*. 1986; Di Silvio *et al*. 1994; Patel *et al*. 1998, 2001*b*; Riggs *et al*. 1999*b*, 2000; Di Silvio *et al*. 2002) and also in the repair of bone and cartilage (Patel & Braden 1991*a*–*c*; Downes *et al*. 1994; Reissis *et al*. 1994*a*,*b*).

**Figure 3:**
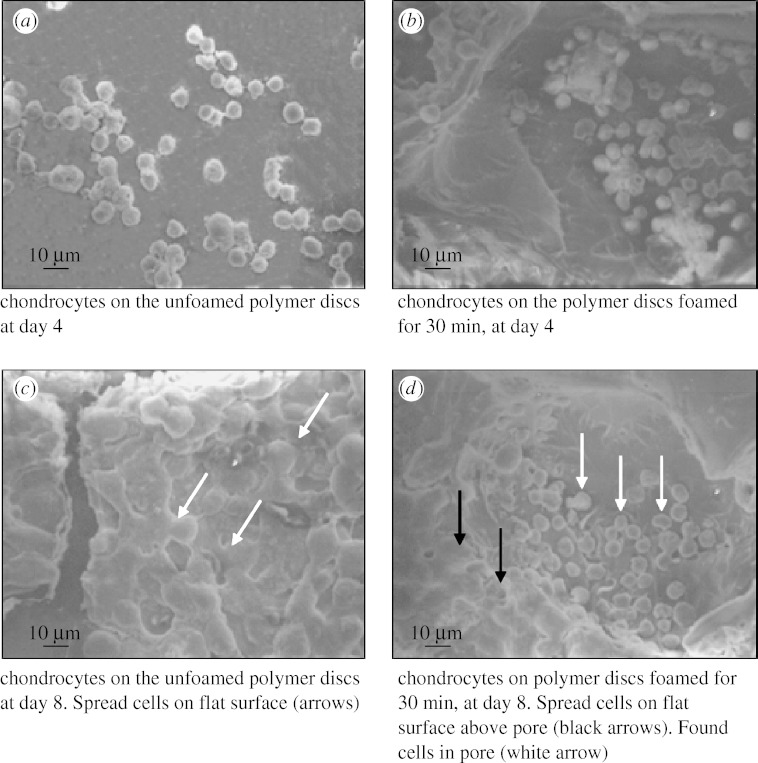
Chondrocytes growing on the unfoamed (*a*,*c*) and foamed (*b*,*d*) PEMA/THFMA substrates. From Barry *et al*. (2004).

Previously, the potential of this polymer system in cartilage repair had been described for two-dimensional surfaces both *in vivo* and *in vitro*. Changing the PEMA/THFMA from a two-dimensional surface to a three-dimensional structure (figure 3) appeared to further enhance the phenotypic maintenance of chondrocytes as indicated by the rounded cell morphology and increased extracellular matrix proteins of articular cartilage (i.e. collagen type II, chondroitin 4-sulphate and chondroitin 6-sulphate; Barry *et al*. 2004). Initial attempts at foaming PEMA/THFMA produced highly porous foams but we were unable to demonstrate connectivity between the pores in the foams. Thus, the emphasis of this work changed and centred on modifying the supercritical processing in order to provide adequate porosity, pore size, pore connectivity and mechanical strength for tissue engineering purposes. This was achieved by decreasing the venting rate.

Decreasing the venting rate permits the nucleation sites to grow into larger pores while also allowing the pores to coalesce, thereby altering pore size and interconnectivity (Barry *et al*. in press *a*). When applied to the PEMA/THFMA scaffolds, the decrease in depressurization rate resulted in highly interconnected porous structures, with mean pore sizes ranging from 100 to 450 μm (measured by SEM). There was, however, an upper limit to these changes in pore architecture beyond which no further benefit can be seen. When this limit is exceeded the pore sizes and mechanical strengths make them unsuitable for use as scaffolds (Barry *et al*. in press *a*).

**Figure 4:**
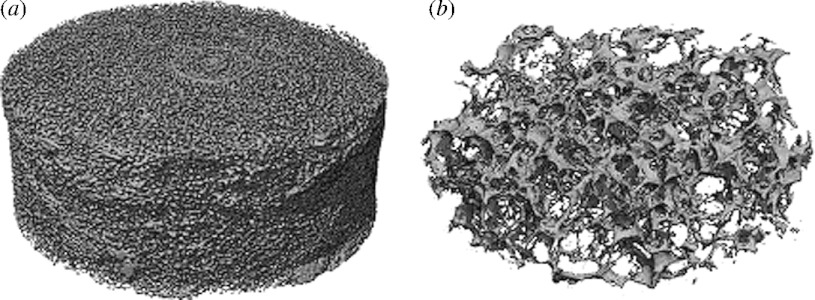
Micro-CT images of scaffolds processed at different rates. (*a*) Rapid depressurization (30 s for 100 bar to ambient pressure). (*b*) Slow depressurization (60 min for 100 bar to ambient pressure). Changes in porosity, pore size and interconnectivity are clearly visible from the images. Images from Barry *et al*. (in press *a*).

This study also highlighted the problems in applying individual techniques to measure the pore size, porosity and interconnectivity (Barry *et al*. in press *a*). For example, while SEM and micro-computed tomography (micro-CT) imaging produced similar results for pore size and pore size distribution, mercury intrusion porosimetry (MIP) generated results that were significantly different. To compensate for this a combination of techniques was applied in order to adequately describe the key characteristics of the scaffolds produced. These techniques include SEM, micro-CT, MIP and helium pycnometry (figure 4).

### (b) SIS/THFMA

The foaming of PEMA/THFMA monoliths leads to a decrease in compliance and ductility, resulting in very brittle scaffolds. As many of the characteristics of the PEMA/THFMA system were attributable to the poly (THFMA) portion an alternative polymeric system based on THFMA was sought. A previous study has investigated the blending of THFMA with an isoprene–styrene copolymer elastomer (SIS) for soft prosthetic applications (Nazhat *et al*. 2001). SIS is an elastomer consisting of ordered block copolymers and has mechanical properties similar to vulcanized natural rubber (Barry *et al*. in press *b*). SIS/THFMA blends produce materials with mechanical properties that are either predominantly plastic or elastomeric whereas the anomalous water uptake behaviour of poly (THFMA) remains unchanged (Barry *et al*. in press *b*).

**Figure 5:**
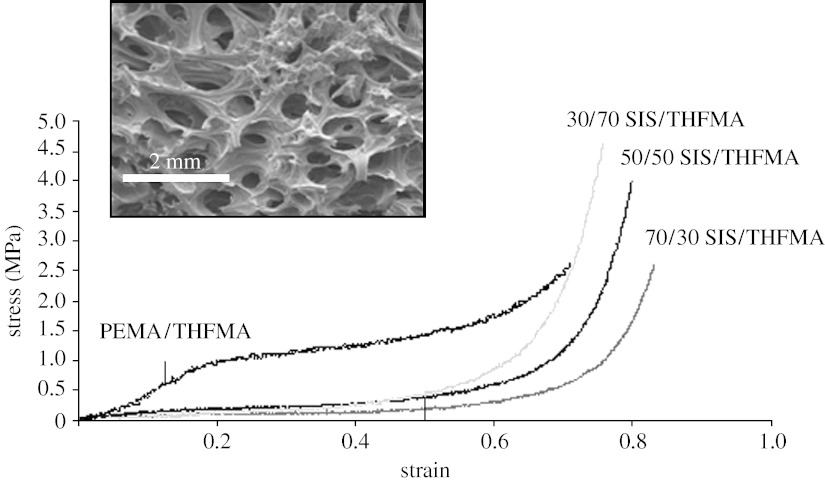
Compressive stress–strain behaviour for SIS/THFMA blend. For comparison, PEMA/THFMA prepared by the same protocol is included. It can be seen that the behaviour of these materials following compression is very different. Damage to the PEMA/THFMA foams occurs at the onset of the plateau and results in densification and ultimately the complete destruction of the foam. This is not the case for the SIS/THFMA, whereby removal of the stress allows the recovery of the material. Inset; electron micrographs of a foamed SIS/THFMA (30%/70%) disc. From Barry *et al*. (in press *b*).

ScCO_2_ was successfully used to foam differing compositions of SIS/THFMA without compromising the materials compliance (figure 5). The swelling of the SIS/THFMA blend was similar to that of figure 2*a–c* in that the monoliths retained their structural integrity throughout the processing until all the CO_2_ had been vented from the high-pressure vessel. However, 24 h after the processing, slight contraction of the foams could be seen. The greater the SIS content in the blend, the smaller the foam that was produced. Similarly, pore size, porosity and interconnectivity all decreased with increasing concentrations of SIS. At low compositions of the SIS, highly porous, interconnected scaffolds (figure 5, *inset*) with mechanical properties that closely match those of cartilage could be produced (Barry *et al*. in press *b*). Subsequent work has shown that these materials like the PEMA/THFMA provide a favourable substrate for chondrocyte attachment and growth.

## 3. Polyester scaffolds

We have utilized scCO_2_ to create polyester scaffolds (e.g. P_L_LA, P_DL_LA, PGA and PLGA) by two methods; the first method is the preparation of PLA monoliths as shown in figure 2*d–f*.

### (a) PLA monoliths

Gas foaming of polyesters was first described in De Ponti *et al*. (1991) and was subsequently followed by the processing of either compressed polyester pellets or solvent cast discs for prolonged periods of time under conditions that are near critical (55 bar at 20–23 °C for 72 h; Mooney *et al*. 1996). Although mistakenly reported as a method that utilizes scCO_2_ (Hutmacher 2000), the prolonged exposure to CO_2_ was long enough to saturate the polymer and upon venting polymeric foams were generated. The foams had pores of 100 μm diameter and porosities up to 93%. Foams produced by this method were found to have low connectivity and a non-porous skin which is formed by rapid loss of CO_2_ from the edge (Goel & Beckman 1994*b*; Mooney *et al*. 1996). The combination of the gas foaming with NaCl particulate leaching did lead to a highly interconnected void network (Sheridan *et al*. 2000; Murphy *et al*. 2002). Control of the porosity and pore size could be achieved by varying the particle/polymer ratio and salt crystal size (Harris *et al*. 1998). Scaffolds fabricated by the combination of gas foaming/salt leaching have been employed both for the controlled delivery of proteins and as a support for the three-dimensional culture of cells (Quirk *et al*. 2004). One drawback of the salt leaching step is that it can remove a large percentage of the bioactive ingredients loaded in the scaffolds (Hutmacher 2000).

**Figure 6:**
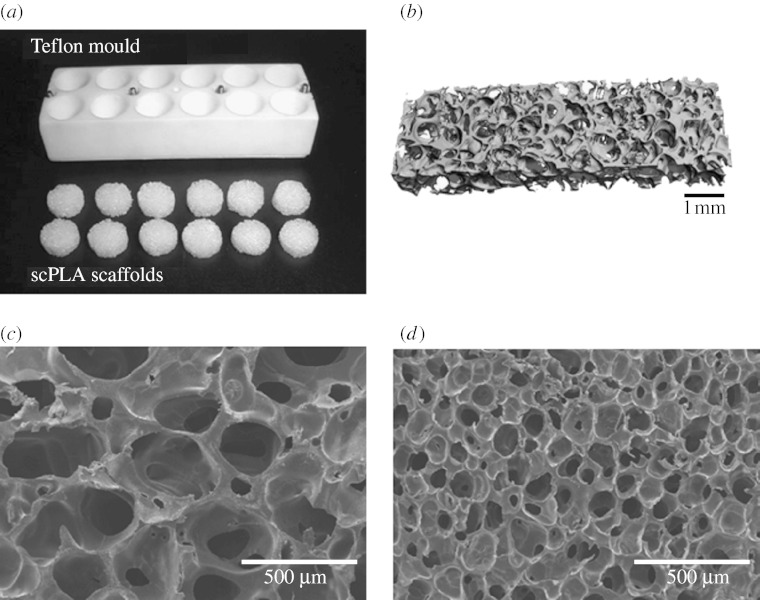
PLA scaffolds. (*a*) The scaffolds as fabricated and removed from the Teflon mould. (*b*) Micro-CT images of the scaffolds, and (*c*,*d*) SEM images demonstrating that variation of temperature, pressure and venting rate leads to scaffolds with different pore architectures. From Quirk *et al*. (2004).

By contrast processing of polyester powders using scCO_2_ has allowed preparation of highly porous, interconnected monoliths in a single step process. As with the methacrylate scaffolds the pore architecture can be modified by temperature, pressure and vent rate (figure 6; Howdle *et al*. 2001). The benefit of this approach is that it allows the incorporation of heat sensitive pharmaceuticals and biological agents into the polyester foams, without loss of the active agents due to the leaching step (Hutmacher 2000). The enzymes ribonuclease A, catalase and β-d-galactosidase have been incorporated into P_DL_LA scaffolds with only negligible loss in biological activity (Howdle *et al*. 2001).

The technique is widely applicable and has been shown to allow the controlled addition of very low concentrations of bioactive guest (Watson *et al*. 2002). Moreover, the fine control of morphology (figure 6) leads to applications as an effective material for bone regeneration. Recent collaborative experiments demonstrate bone formation due to the release of the osteoinductive protein bone morphogenetic factor 2 (BMP-2) from P_DL_LA scaffold both *in vitro* and *in vivo* (Yang *et al*. 2001*a*, Yang *et al*. 2003*a*,*b*). These scaffolds have further been used to study adenoviral gene transfer into primary human bone marrow osteoprogenitor cells (Partridge *et al*. 2002; Howard *et al*. 2002). Recently, it has been demonstrated that the surfaces of these scCO_2_ prepared scaffolds can be made more adhesive by plasma deposition of a fine layer of poly(allylamine). This was achieved without changing the bulk characteristics of the polymer and significantly increased cell attachment on the otherwise hydrophobic materials (Barry *et al*. 2005).

### (b) Particle generation

**Figure 7:**
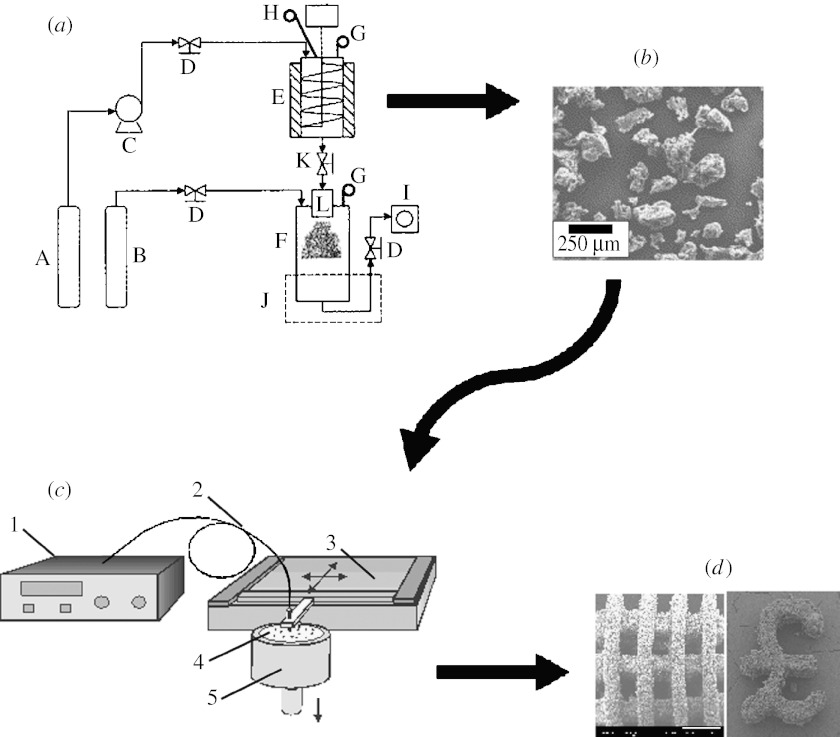
(*a*) Schematic diagram for the supercritical fluid apparatus used to prepare P_DL_LA particles: A, CO_2_ cylinder; B, nitrogen cylinder; C, CO_2_ feed pump; D, valves; E, mixing vessel; F, collecting chamber; G, pressure gauges; H, thermocouple; I, backpressure regulator; J, cooling device; K, spraying (ball) valve; L, cone nozzle (Hao *et al*. 2004). (*b*) SEM images of P(_DL_LA) particles (Whitaker *et al*. 2005). (*c*) Schematic diagram of experimental SLS set-up. 1, diode laser; 2, quartz fibre; 3, *X–Y* plotter; 4, polymer powder; 5, powder container with precision elevator piston (*Z*-axis) (Antonov *et al*. 2005). (*d*) PLA scaffold made using the sintering process described in text.

The formation of a polymer/gas solution by supercritically plasticized polymers is seen in figure 2*e*. If this solution is forced through a nozzle during depressurization, fine microparticles can be fabricated (figure 7*a*,*b*; Hao *et al*. 2004). The size and shape of these microparticles can be finely controlled by altering the nozzle diameter, the saturation temperature or filling the collecting chamber with N_2_ gas. The N_2_ backpressure dynamically regulates the loss of CO_2_ from the issuing polymer/CO_2_ mixture (Hao *et al*. 2004).

These particles can be used in drug delivery as temperature and solvent labile molecules can be mixed efficiently into the liquefied polymer. This liquefied polymer/drug/CO_2_ mixture can then be sprayed into a collecting chamber, and during this process particles of drug-loaded polymer are formed, which can be used for drug delivery (Whitaker *et al*. 2005). Growth factor loaded microparticles such as these are also finding applications as injectable scaffolds that can be delivered to specific sites within the body for cell and tissue repair (Salem *et al*. 2003).

Recently, these microparticles have been used to fabricate scaffolds using a novel *Surface* Selective Laser Sintering (*S*SLS) process (figure 7*c*; Antonov *et al*. 2005). In conventional SLS, volumetric absorption of the laser radiation by polymer leads to melting of the whole particles followed by their fusion. Exposure of the polymer powders to high temperatures is prohibitive to many thermoliable materials. In SSLS, this particle fusion can be limited to just the surfaces of each particle. This is achieved by use of near infrared (*ca* 1 μm) laser wavelength, which is not absorbed by the polymer and by the addition of a small amount (less than 0.1 wt%) of carbon microparticles. The carbon microparticles are strong absorbers of the laser radiation, hence they cause heating of only the surfaces of each polymer particle. Thus, each particle fuses with its neighbour, but without overheating and or collapse of the particle structure. This method therefore facilitates the sintering of polymers that were previously unsuitable for SLS. For example, P_DL_LA can now be used to fabricate complex three-dimensional structures such as scaffolds (figure 7*d*). Additionally, if the particles are first loaded with growth factors or drugs via the supercritical route, then this process allows the fabrication of bioactive/biodegradable scaffolds in which the pore sizes and porosity, connectivity and orientation of the pores can be finely controlled (Antonov *et al*. 2005). Moreover, because there is only surface heating of the particles, the SLS process allows retention of the activity of the bioactive. This has been demonstrated (Antonov *et al*. 2005) by the encapsulation of a model protein, (ribonuclease A) into particles of poly(d,l-lactic) acid (PLA) and the fabrication of three-dimensional scaffolds via SLS.

## 4. Conclusion

This paper has briefly reviewed some of the recent progress made in the use of supercritical fluids to process polymers into three-dimensional tissue engineering scaffolds. It has described the plasticization and foaming of hydrophilic methacrylate polymer blends to produce tissue engineering scaffolds. Control of the pore architecture in these scaffolds is achieved by altering the foaming conditions as has been shown also for the polyester scaffolds.

For polyesters, the polymer viscosity is lower dramatically, leading to efficient mixing of bioactive materials and fabrication of three-dimensional scaffolds, which can be used to enhance tissue regeneration. The same methodology can be adapted to prepare particles of biodegradable polymer also loaded with bioactive molecules. Such particles have proved to be very effective in a surface selective laser sintering process leading to novel three-dimensional scaffolds with precise shape and porosity. In all of these cases, the use of scCO_2_ provides a clean and extremely effective route to novel three-dimensional scaffolds that would be difficult or impossible to fabricate through more conventional technologies.

## References

[bib13] Alavi S.H. Gogoi B.K. Khan M. Bowman B.J. Rizvi S.S.H. Food Res. Int 32 1999 107–118. 10.1016/S0963-9969(99)00063-0.

[bib57] Antonov E.N. Bagratashvili V.N. Whitaker M.J. Barry J.J.A. Shakesheff K.M. Konovalov A.N. Popov V.K. Howdle S.M. Adv. Mater 17 2005 327–330. 10.1002/adma.200400838. PMC1855444 17464361

[bib12] Arora K.A. Lesser A.J. McCarthy T.J. Macromolecules 31 1998 4614–4620. 10.1021/ma971811z.

[bib41] Barry J.J.A. Gidda H.S. Scotchford C.A. Howdle S.M. Biomaterials 25 2004 3559–3568. 10.1016/j.biomaterials.2003.10.023. 15020130

[bib54] Barry J.J.A. Silva M.M.C.G. Shakesheff K.M. Howdle S.M. Alexander M.R. Adv. Funct. Mater 15 2005 1134–1140. 10.1002/adfm.200400562.

[bib25] Barry, J. J. A., Silva M. C. G. S., Cartmell S. H., Guldberg R. E., Scotchford C. A. & Howdle S. M. In press *a* *J. Mater. Sci.: Mater. Med*.

[bib42] Barry, J. J. A., Nazhat, S. N., Scotchford C. A. & Howdle S. M. In press *b* *J. Mater. Chem*.

[bib27] Bhusate, M. & Braden, M. 1985 Patent GB 2 107 341 A, UK.

[bib6] Cooper A.I. J. Mater. Chem 10 2000 207–234. 10.1039/a906486i.

[bib43] De Ponti, R., Torricelli, C., Martini, A. & Lardini, E. 1991 Patent PCT/EP90/01895.

[bib30] Di Silvio L. Kayser M.V. Downes S. Clin. Mater 16 1994 91–98. 10.1016/0267-6605(94)90102-3. 10147328

[bib31] Di Silvio L. Dalby M.J. Bonfield W. Biomaterials 23 2002 101–107. 10.1016/S0142-9612(01)00084-9. 11762828

[bib35] Downes S. Archer R.S. Kayser M.V. Patel M.P. Braden M. J. Mater. Sci.: Mater. Med 5 1994 88–95. 10.1007/BF00121696.

[bib7] Goel S.K. Beckman E.J. Polym. Eng. Sci 34 1994a 1137–1148. 10.1002/pen.760341407.

[bib16] Goel S.K. Beckman E.J. Polym. Eng. Sci 34 1994b 1148–1156. 10.1002/pen.760341408.

[bib2] Goldstein S.A. Moalli M.R. Curr. Opin. Orthop 12 2001 424–427. 10.1097/00001433-200110000-00010.

[bib55] Hao J. Whitaker M.J. Wong B. Serhatkulu G. Shakesheff K.M. Howdle S.M. J. Pharm. Sci 93 2004 1083–1090. 10.1002/jps.20002. 14999744

[bib46] Harris L.D. Kim B.S. Mooney D.J. J. Biomed. Mater. Res 42 1998 396–402. 10.1002/(SICI)1097-4636(19981205)42:3%3C396::AID-JBM7%3E3.0.CO;2-E. 9788501

[bib53] Howard D. Partridge K. Yang X. Clarke N.M.P. Okubo Y. Bessho K. Howdle S.M. Shakesheff K.M. Oreffo R.O.C. Biochem. Biophys. Res. Commun 299 2002 208–215. 10.1016/S0006-291X(02)02561-5. 12437971

[bib10] Howdle S. M. 2004 In http://www.nottingham.ac.uk/∼pczctg/Index2.htm.

[bib15] Howdle S.M. Watson M.S. Whitaker M.J. Popov V.K. Davies M.C. Mandel F.S. Wang J.D. Shakesheff K.M. Chem. Commun 01 2001 109–111. 10.1039/b008188o.

[bib20] Hutcheon G.A. Downes S. Davies M.C. J. Mater. Sci.: Mater. Med 9 1998 815–818. 10.1023/A:1008940027063. 15348946

[bib44] Hutmacher D.W. Biomaterials 21 2000 2529–2543. 10.1016/S0142-9612(00)00121-6. 11071603

[bib18] Lee S.Y. Loh K.Y. Leong L.S. Goh S.H. Eur. Polym. J 28 1992 229–232. 10.1016/0014-3057(92)90180-A.

[bib4] Leong K.F. Cheah C.M. Chua C.K. Biomaterials 24 2003 2363–2378. 10.1016/S0142-9612(03)00030-9. 12699674

[bib3] Ma P.X. Mater. Today 7 2004 30–40. 10.1016/S1369-7021(04)00233-0.

[bib8] McHugh M.A. Krukonis V.J. Supercritical fluid extraction: principles and practice. In Butterworth-Heinemann series in chemical engineering 2nd edn. 1994 Boston:Butterworth-Heinemann

[bib11] Mooney D.J. Baldwin D.F. Suh N.P. Vacanti J.P. Langer R. Biomaterials 17 1996 1417–1422. 10.1016/0142-9612(96)87284-X. 8830969

[bib45] Murphy W.L. Dennis R.G. Kileny J.L. Mooney D.J. Tissue Eng 8 2002 43–52. 10.1089/107632702753503045. 11886653

[bib24] Nazhat S.N. Parker S. Patel M.P. Braden M. Biomaterials 22 2001 2411–2416. 10.1016/S0142-9612(00)00428-2. 11511038

[bib17] Park C.B. Baldwin D.F. Suh N.P. Polym. Eng. Sci 35 1995 432–440. 10.1002/pen.760350509.

[bib52] Partridge K. Yang X. Clarke N.M.P. Okubo Y. Bessho K. Sebald W. Howdle S.M. Shakesheff K.M. Oreffo R.O.C. Biochem. Biophys. Res. Commun 2921 2002 144–152. 10.1006/bbrc.2002.6623. 11890685

[bib38] Patel M.P. Braden M. Biomaterials 12 1991a 645–648. 10.1016/0142-9612(91)90110-V. 1742407

[bib39] Patel M.P. Braden M. Biomaterials 12 1991b 649–652. 10.1016/0142-9612(91)90111-M. 1742408

[bib40] Patel M.P. Braden M. Biomaterials 12 1991c 653–657. 10.1016/0142-9612(91)90112-N. 1742409

[bib19] Patel M.P. Braden M. Davy K.W. Biomaterials 8 1987 53–56. 10.1016/0142-9612(87)90030-5. 3828447

[bib32] Patel M.P. Pearson G.J. Braden M. Mirza M.A. Biomaterials 19 1998 1911–1917. 10.1016/S0142-9612(98)00048-9. 9863524

[bib23] Patel M.P. Johnstone M.B. Hughes F.J. Braden M. Biomaterials 22 2001a 81–86. 10.1016/S0142-9612(00)00171-X. 11085387

[bib34] Patel M.P. Cruchley A.T. Coleman D.C. Swai H. Braden M. Williams D.M. Biomaterials 22 2001b 2319–2324. 10.1016/S0142-9612(00)00367-7. 11511028

[bib29] Pearson G.J. Picton D.C. Braden M. Longman C. Int. Endod. J 19 1986 121–124. 3460966 10.1111/j.1365-2591.1986.tb00464.x

[bib9] Quirk R. A., France R. M., Shakesheff K. M. & Howdle S. M. 2004 *Curr. Opin. Solid State Mater. Sci* **8**, 313–321.

[bib36] Reissis N. Downes S. Kayser M. Lee D. Bentley G. J. Mater. Sci.: Mater. Med 5 1994a 402–406. 10.1007/BF00058973.

[bib37] Reissis N. Downes S. Kayser M. Lee D. Bentley G. J. Mater. Sci.: Mater. Med 5 1994b 793–797. 10.1007/BF00213137.

[bib21] Riggs P.D. Braden M. Tilbrook D.A. Swai H. Clarke R.L. Patel M.P. Biomaterials 20 1999a 435–441. 10.1016/S0142-9612(98)00188-4. 10204986

[bib33] Riggs P.D. Braden M. Tilbrook D.H. Swai H. Clarke R.L. Patel M.P. Biomaterials 20 1999b 435–441. 10.1016/S0142-9612(98)00188-4. 10204986

[bib28] Riggs P.D. Braden M. Patel M.P. Biomaterials 21 2000 345–351. 10.1016/S0142-9612(99)00187-8. 10656315

[bib1] Sachlos E. Czernuszka J.T. Eur. Cells Mater 5 2003 29–40. 10.22203/ecm.v005a03 14562270

[bib58] Salem A.K. Adv. Mater 15 2003 210–213. 10.1002/adma.200390047.

[bib22] Sawtell R.M. Downes S. Patel M.P. Clarke R.L. Braden M. J. Mater. Sci.: Mater. Med 8 1997 667–674. 10.1023/A:1018531722265. 15348817

[bib14] Sheridan M.H. Shea L.D. Peters M.C. Mooney D.J. J. Control. Release 64 2000 91–102. 10.1016/S0168-3659(99)00138-8. 10640648

[bib48] Watson M.S. Whitaker M.J. Howdle S.M. Shakesheff K.M. Adv. Mater 14 2002 1802–1804. 10.1002/adma.200290003.

[bib56] Whitaker M.J. Hao J. Davies O.R. Serhatkulu G. Stolnik-Trenkic S. Howdle S.M. Shakesheff K.M. J. Control. Release 101 2005 85–92. 10.1016/j.jconrel.2004.07.017. 15588896

[bib5] Woods H.M. Silva M.M.C.G. Nouvel C. Shakesheff K.M. Howdle S.M. J. Mater. Chem 14 2004 1663–1678. 10.1039/b315262f.

[bib49] Yang X.B. Roach X.I. Clarke N.M. Howdle S.M. Quirk R. Shakesheff K.M. Oreffo R.O.C. Bone 29 2001 523–531. 10.1016/S8756-3282(01)00617-2. 11728922

[bib50] Yang X.B. Tare R.S. Partridge K.A. Roach H.I. Clarke N.M. Howdle S.M. Shakesheff K.M. Oreffo R.O.C. J. Bone Miner. Res 18 2003a 47–57. 12510805 10.1359/jbmr.2003.18.1.47

[bib51] Yang X.B. Green D.W. Roach H.I. Clarke N.M. Anderson H.C. Howdle S.M. Shakesheff K.M. Oreffo R.O.C. Connect. Tissue Res 44 Suppl. 1 2003b 312–317. 12952215

